# Smartphone dependence predicts poorer mental health outcomes, eating behaviors, activity levels, and body image: a cluster analysis of Brazilian university students

**DOI:** 10.47626/2237-6089-2024-0904

**Published:** 2025-09-09

**Authors:** Karen Rodrigues Lima, Bárbara Isabela Amorim, Débora Ribeiro Orlando, Luciano José Pereira, Paula Midori Castelo, Eric Francelino Andrade

**Affiliations:** 1 Universidade Federal de Lavras Departamento de Ciências da Saúde Lavras MG Brazil Departamento de Ciências da Saúde, Universidade Federal de Lavras, Lavras, MG, Brazil.; 2 Universidade Federal de São Paulo Departamento de Ciências Farmacêuticas Diadema SP Brazil Departamento de Ciências Farmacêuticas, Universidade Federal de São Paulo, Diadema, SP, Brazil.

**Keywords:** Internet addiction disorder, feeding and eating disorders, body image, anxiety, depression

## Abstract

**Objective:**

Excessive smartphone use has been linked to mental health impairments and may potentially alter human behavior. These effects are particularly pronounced among younger individuals, with university students being especially susceptible to the negative influences of smartphone use.

**Methods:**

This observational, cross-sectional study was conducted in a sample of 781 Brazilian university students. We assessed socio-economic variables, smartphone addiction, depression, anxiety, stress outcomes, eating behavior, body image satisfaction, and self-reported physical activity. Multivariate analysis of variance (MANOVA) and chi-square tests were performed to compare continuous and categorical variables between genders. K-means clustering was used to identify participant profiles based on various self-reported variables, with differences between clusters validated using the Z-test and the silhouette coefficient.

**Results:**

Three clusters were identified. Cluster 1 featured participants with a significant disparity between their perceived and desired body image, higher scores on eating disorders, smartphone addiction, and mental health questionnaires, and lower levels of physical exercise. Cluster 2 consisted of older participants who scored lower for smartphone addiction and mental health and had a higher body mass index (BMI). Cluster 3 included younger participants with a smaller Silhouette Scale disparity, lower eating disorder scores, and lower BMI. Smartphone addiction showed significant associations with eating disorders in the overall eating disorders classification (χ² = 13.4; p < 0.001), bulimic behavior (χ² = 20.0; p < 0.001), and social pressure to eat (χ² = 4.3; p < 0.001). It also negatively correlated with physical exercise (χ² = 5.7; p = 0.017), but not with dieting concerns (χ² = 0.23; p = 0.688).

**Conclusion:**

Smartphone addiction is associated with eating disorders, stress, depression, anxiety, and lower levels of physical activity.

## Introduction

Smartphones are considered the major screen device used in modern times.^1^ These devices facilitate quick research, communication, and online social interaction.^2^ However, despite these advantages, excessive smartphone use has garnered attention due to its potential health detriments.^3^ Nomophobia is a term used to describe the fear and discomfort caused by the lack of contact with or access to a smartphone and/or the internet, which is a phenomenon that can lead to mood changes, depression, anxiety, social phobia, and other issues.^4^

Smartphone dependence has not yet been recognized as a disorder in psychiatric manuals and receives less attention compared to substance use disorders.^5^ There is ongoing debate in the literature about whether this behavior should be classified as an addiction, as its impact on functional impairment may not reach the severity levels associated with other addictions.^6^ Additionally, there is currently no gold standard for diagnostic criteria, which may create a potential for misclassification of individuals as "smartphone addicted."^7^ Therefore, the condition is more accurately described as "problematic use" or "dependence."^6^ Smartphone dependence occurs when individuals become so immersed in smartphone use that they neglect other areas of their lives.^8^ Thus, this dependency is considered a behavioral issue that affects aspects of cognitive, social, and psychological development, particularly in young individuals.^9–11^ Additionally, eating and body image disorders are behavioral aspects that may be associated with smartphone dependence.^12^ Excessive smartphone use can also lead to unhealthy eating behaviors, such as the consumption of fast food and junk food, and can negatively impact physical activity.^12^

University students are prone to smartphone addiction and psychological disorders such as anxiety and depression.^13,14^ However, behavioral, environmental, and cultural factors can influence the relationship between smartphone addiction and behavioral and mental health outcomes.^15^ Therefore, assessing these parameters in students from different countries could provide valuable information to aid in the development of interventions aimed at reducing the negative impacts on mental health and quality of life. Thus, we aim to evaluate the relationships between smartphone addiction and mental health outcomes (anxiety, depression, and stress), eating behavior, body image satisfaction, and physical activity among Brazilian university students.

## Materials and methods

### Sample

The sample comprised 781 Brazilian university students (410 males and 371 females) enrolled at both public and private institutions. Participants completed an online survey prepared on Google Forms (Alphabet, Mountain View, CA, USA). The survey included a socio-economic questionnaire that collected information on age, gender, marital status (both participant and parents), educational level (both participant and parents), physical activity level, prior diagnoses of anxiety and depression, and use of psychotropic medications. Additionally, self-reported anthropometric data on weight and height were collected to calculate body mass index (BMI). Furthermore, participants also completed the Smartphone Addiction Inventory (SPAI-BR), the Depression, Anxiety and Stress Scales (DASS-21), and the Silhouette Scale, as well as the Eating Attitude Test-40 (EAT-40). Invitations to participate were sent through social networks including Facebook^®^, Instagram^®^, and WhatsApp^®^. An online snowball sampling strategy was employed to recruit as many respondents as possible.

All participants voluntarily provided informed consent online to participate in the study. The exclusion criteria included individuals under 18 years of age, non-students, non-Brazilian Portuguese speakers, and those who did not complete the entire online survey.

### Instruments

Smartphone dependence was assessed using the Brazilian version of the SPAI-BR.^16^ This instrument addresses symptoms of withdrawal, excessive usage time, and interference with daily activities. The SPAI-BR consists of 26 dichotomous items (response options are yes and no). A score above 10 points, out of a total of 26 points, was considered indicative of smartphone dependence.^17^

The Brazilian version of the DASS-21 was used to evaluate participants’ levels of depression, anxiety, and stress.^18^ This instrument consists of 21 items, each rated on a scale from 0 ("Strongly Disagree") to 3 ("Totally Agree"). Each of the three subscales (depression, anxiety, and stress) contains seven items and the final classification for each subscale is obtained by summing the respective item scores, with scores of ≥ 10, ≥ 8, and ≥ 15 indicating positive classifications for depression, anxiety, and stress respectively.^18^

Body image satisfaction was assessed using the Silhouette Scale for Adults.^19^ To determine body image (dis)satisfaction, the difference between participants’ perceived silhouette and their desired silhouette was calculated according to the guidelines of the original instrument.^19^

Eating behavior aspects were assessed using the EAT-40,^20^ a tool designed to identify the risk of eating disorders. This instrument consists of 40 questions with Likert scale responses ranging from 0 (never) to 5 (always). The response options are inverted for six items (1, 18, 19, 23, 27, and 39). The total score ranges from 0 to 200 and is obtained by summing the responses, with higher scores indicating more dysfunctional eating behaviors. Additionally, the instrument can identify specific domains of eating behavior: "dieting concern," "bulimic behavior," and "social pressure to eat."^21^

### Statistical analysis

Statistical analysis was conducted using SPSS 28.0 and Past4 (PMC) software. Exploratory analysis included calculation of means, standard deviations (SD), medians, and percentages and graphical analysis. A significance level of 5% was adopted. Comparison of continuous variables between genders was performed using multivariate analysis of variance (MANOVA), and associations with categorical variables were tested using the chi-square test.

Cluster analysis (K-means clustering) was employed to identify participant profiles with similar variables related to self-reported aspects of smartphone addiction, eating disorders, anxiety, depression, stress, physical exercise, and BMI. This analysis method is highly useful for understanding the complex nature of multivariate relationships. The following variables were included: age, gender, Silhouette Scale difference, EAT-40, SPAI-BR, and DASS-21 scores, physical exercise, and BMI. Differences between clusters were described using the Z-test for validation, and test consistency assessment was based on the silhouette coefficient.

### Ethical considerations

The study was approved by the Universidade Federal de Lavras Research Ethics Committee under protocol number 5.379.531.

## Results

**Figure 1:**
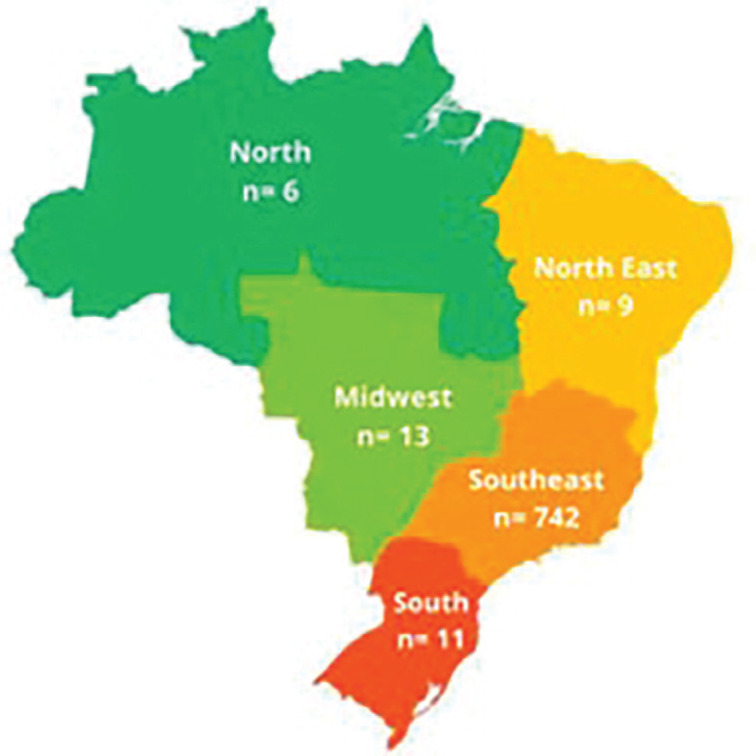
Numbers of participants by region of Brazil.

Table 1 displays the clinical and demographic characteristics of participants categorized by gender. There were no differences in age, EAT-40, SPAI-BR, DASS-21, or Silhouette Scale scores between genders (p > 0.05). The distribution of participants by geographical region is illustrated in Figure 1.

**Table 1:** Demographic and clinical characteristics of participants by gender (n = 781)

Characteristics		Male (n = 410)	Female (n = 371)
Age, years			
	Mean (SD)	24.3 (6.7)	24.7 (7.4)
	Min-max	16-65	17-65
		
Marital status (%)			
	Divorced/separated/single	30.5	30.6
	Married/common-law marriage	69.5	69.4
		
Parental marital status during childhood (%)			
	Married/common-law marriage	79.8	80.3
	Separated/divorced	11.0	11.9
	Did not grow up with my father/mother	3.3	3.4
		
Field of knowledge (%)			
	Biomedical sciences	34.9	19.7
	Agricultural sciences	14.9	4.3
	Applied social sciences	21.5	38.8
	Exact and earth sciences/engineering	21.2	26.4
	Humanities, linguistics, literature, and arts	7.6	10.8
		
Anxiety/depression diagnosis (yes) (%)		52.7	52.8
		
Physical exercise practice (yes), times per week (%)		57.8	58.5
	1	27.8	35.6
	2-4	39.5	39.4
	5-7	21.7	21.8
		
BMI classification* (%)			
	Underweight	7.6	5.8
	Normal weight	59.3	56.7
	Overweight	22.5	22.8
	Obesity	10.6	14.6
		
Actual body image (median [25-75%])		7.0 (6-10)	8.0 (5-10)
Desired body image (median [25-75%])		6.0 (5-7)	6.0 (5-7)
Difference between actual and desired image (median [25-75%])		1.5 (0-3)	2.0 (0-3)

SD = standard deviation.

* Self-report; contains missing data.

It was observed that 271 participants (34.6%) were classified as having a smartphone addiction, based on the cutoff specified in the Sample subsection of the Materials and methods section (a score of 10 or higher out of 26 on the SPAI-BR). The relative frequency of this condition was higher among women compared to men (Table 2). The EAT-40 total scores classified 117 participants (14.9%) as having dysfunctional eating behavior, with 29 (3.7%) scoring positive for dieting concern, 122 (15.6%) classified with bulimic behavior, and 18 (2.3%) having high scores for social pressure to eat. On the basis of the DASS-21 results, it was observed that 294 (37.6%) participants were positive for depression, while 327 (41.8%) and 356 (45.5%) were classified as having anxiety and stress, respectively. Table 2 presents the frequency data of participants by sex for positive classifications according to the SPAI-BR, EAT-40, and DASS-21.

**Table 2:** Participant characteristics by sex according to scores on the Smartphone Addiction Inventory (SPAI-BR), Eating Attitude Test-40 (EAT-40), and Depression, Anxiety and Stress Scales (DASS-21)

Instrument	Classification/domain	Men (n = 410)	Women (n = 371)
SPAI-BR	-	105 (25.6)	167 (45.0)
EAT-40	Dieting concern	16 (3.9)	13 (3.5)
	Bulimic behavior	69 (16.8)	53 (14.3)
	Social pressure to eat	13 (3.1)	5 (1.3)
	Total	66 (16.1)	51 (13.7)
DASS-21	Depression	159 (38.7)	135 (36.3)
	Anxiety	180 (43.9)	147 (39.6)
	Stress	189 (46.1)	167 (45.0)

Data presented as n (%).

Cluster analysis identified three distinct profiles among the participants that met the criteria for interpretability, varying according to age, body image, self-reported eating disorders, BMI, physical exercise practice, symptoms of depression/anxiety/stress, and smartphone addiction, considering the parameter of the Z-test conducted. Table 3 shows that Cluster 1, termed "Smartphone Addiction," included participants with a greater discrepancy between their perceived and desired body image (Silhouette Scale), higher scores on the EAT-40, SPAI-BR, and DASS-21 questionnaires, and a lower frequency of positive responses for physical exercise practice. Cluster 2 (Older Age and Lower Smartphone Addiction) included older participants (average age of 43 years) who scored lower on the SPAI-BR and DASS-21 questionnaires and had higher BMI. Finally, Cluster 3 (Fewer Eating Disorders) comprised younger participants with a smaller discrepancy on the Silhouette Scale, lower scores on the EAT-40 instrument, and lower BMI.

**Table 3:** Description of the groups (clusters) generated from the study variables (centroids; means)

	Cluster 1:	Cluster 2:	Cluster 3:	Z-test	ANOVA p-value
	Smartphone addiction	Older age and lower smartphone addiction	Fewer eating disorders		
n	211	58	512		
Age	23.2	**43.9**	**22.8**	602.6	< 0.001
Sex	0.5	0.6	0.5	1.6	0.211
Body image*	**2.7**	2.0	**0.7**	43.4	< 0.001
EAT-40	**31.2**	16.7	**15.9**	397.8	< 0.001
SPAI-BR	**14.4**	**7.6**	10.2	64.2	< 0.001
DASS-21 depression	**13.7**	**3.8**	6.2	189.2	< 0.001
DASS-21 anxiety	**12.0**	**3.2**	4.8	205.4	< 0.001
DASS-21 stress	**15.1**	**6.2**	8.4	178.0	< 0.001
Physical activity	**0.6**	0.7	0.7	4.4	0.012
BMI	24.4	**27.9**	**23.5**	14.4	< 0.001

ANOVA = analysis of variance; BMI = body mass index; DASS-21 = Depression, Anxiety and Stress Scales; EAT-40 = Eating Attitude Test-40; SPAI-BR: Smartphone Addiction Inventory.

Differences that define the clusters are highlighted in bold type.

* Silhouette Scale coefficient = 0.30.

Indeed, a more detailed analysis revealed that smartphone addiction was significantly associated with the presence of eating disorders in the overall EAT-40 classification (χ² = 13.4; p < 0.001), as well as in the domains of bulimic behavior (χ² = 20.0; p < 0.001) and social pressure to eat (χ² = 4.3; p < 0.001). Smartphone addiction was also negatively associated with physical exercise (χ² = 5.7; p = 0.017), but not with concern about dieting (χ² = 0.23; p = 0.688).

## Discussion

The main findings of this study include the definition of participant profiles based on sociodemographic and clinical characteristics, resulting in the identification of three clusters: "smartphone addiction," "older age and lower smartphone addiction," and "fewer eating disorders." Through cluster analysis, it was possible to identify that those participants with higher smartphone dependency exhibited greater body image distortion and a higher risk of developing eating and psychological disorders (anxiety, depression, and stress), in addition to less physical exercise practice. Additionally, the study also identified significant associations between smartphone addiction and overall EAT-40 scores, as well as with bulimic behavior and social pressure to eat. Another noteworthy finding was the negative association between smartphone addiction and self-reported physical activity.

Cluster analysis is a highly effective tool for identifying patterns within specific groups based on sociodemographic and behavioral characteristics.^22^ Additionally, this analysis provides a method for screening of mental health indicators, serving as a crucial starting point for establishing therapeutic and preventive strategies by considering the determinants of specific issues.^23^ As found in the present study, higher smartphone dependency has been associated with poorer mental health outcomes previously.^24^ In a study conducted with nursing students, it was reported that anxiety and depression levels were higher among participants classified as having smartphone addiction.^24^ Smartphone addiction is believed to have a bidirectional relationship with anxiety and depression, with nomophobia being a risk factor for developing these disorders.^25^ Conversely, individuals with anxiety and depression are more prone to becoming dependent on their devices.^25^

Considering the profile of individuals in Cluster 1, greater discrepancies were observed between the current silhouette and the desired silhouette, indicating higher body image dissatisfaction. Body image dissatisfaction is a common trait among anxious and depressed individuals,^26^ aligning with the characteristics observed in the present study. Additionally, smartphone dependency has been associated with body image distortion among Korean adolescents.^26^ This relationship may be explained by the content accessed on these devices by younger individuals, who primarily use them to access social media.^27^ Social media platforms often promote idealization of an ideal body type, with thinness for women and muscularity for men.^27,28^ Thus, even though this relationship is more evident in adolescents,^26–28^ young adults may also exhibit these behaviors, influencing their body image satisfaction as observed in the present study.

Regarding eating behavior, in our study, individuals located in Cluster 1 exhibited higher scores on the EAT-40, which were associated with increased anxiety, depression, body dissatisfaction, and smartphone dependency. Additionally, Cluster 3 (Fewer eating disorders) included participants with lower scores on eating disorder measures, lower body dissatisfaction, and lower BMI values. The behavior observed in these clusters is consistent, given that body dissatisfaction is a predominant factor in eating disorders.^26^

To the best of our knowledge, few studies have explored the relationship between smartphone addiction and higher scores in the domains of bulimic behavior and social pressure to eat. Previous studies have reported similar findings regarding the association between smartphone addiction and overall scores on the EAT-40.^3,28^ Among university students, it has been observed that greater internet and smartphone usage correlates with higher EAT-40 scores.^2^ Additionally, students with smartphone dependence have shown higher scores on the abbreviated version of the EAT (EAT-26).^3^ It is described in the literature that individuals with internet addiction are more likely to meet the Diagnostic and Statistical Manual of Mental Disorders, 4th edition (DSM-IV) criteria for bulimia, with depression considered as a mediator in this relationship.^29^ Furthermore, social media addiction has also been weakly associated with increased risk of developing bulimia.^30^ These prior findings support the results of the present study, as smartphones are now the primary means of internet access for most individuals.

Regarding the association between smartphone addiction and higher scores in the domain of "social pressure to eat," it is conceivable that because a substantial portion of smartphone use revolves around accessing social networks, young individuals are exposed to comments and criticisms regarding their bodies, thereby feeling compelled to meet thinness standards.^31,32^

One interesting finding of the present study was the negative association between smartphone addiction and physical exercise. While the content accessed on smartphones can serve as a motivator for physical activity, smartphone addiction appears to increase sedentary behavior (e.g., prolonged sitting) and reduce the quality and quantity of moderate and vigorous physical activities.^33,34^ A similar trend was observed in a sample of Chinese students, where engaging in physical activity was considered a protective factor against the onset of smartphone dependency.^35^

## Conclusion

Based on the results observed in the present study, we conclude that smartphone addiction is associated with eating disorders, stress, depression, anxiety, and lower levels of physical activity. These findings underscore the need for interventions aimed at limiting screen time among university students, as these outcomes could negatively impact quality of life and academic performance. Future studies should include an investigation of the content accessed and explore whether there is a relationship between this content and other behavioral and mental health outcomes.

## Data Availability

The data that support this study are available from the authors upon request.
